# MiR-146b-5p enriched bioinspired exosomes derived from fucoidan-directed induction mesenchymal stem cells protect chondrocytes in osteoarthritis by targeting TRAF6

**DOI:** 10.1186/s12951-023-02264-9

**Published:** 2023-12-18

**Authors:** Chao Lou, Hongyi Jiang, Zhongnan Lin, Tian Xia, Weidan Wang, Chihao Lin, Zhiguang Zhang, Haonan Fu, Shoaib Iqbal, Haixiao Liu, Jian Lin, Jilong Wang, Xiaoyun Pan, Xinghe Xue

**Affiliations:** 1 Department of Orthopedics, The Second Affiliated Hospital, Yuying Children’s Hospital of Wenzhou Medical University, Wenzhou, Zhejiang Province China; 2 Key Laboratory of Orthopedics of Zhejiang Province, Wenzhou, Zhejiang Province China; 3 Wenzhou Institute, University of Chinese Academy of Sciences, Wenzhou, Zhejiang Province China; 4 Feik School of Pharmacy, University of the Incarnate Word, Broadway, San Antonio, 4301 USA

**Keywords:** Osteoarthritis, Exosomes, Fucoidan, MSCs, miR-146b-5p, TRAF6

## Abstract

Osteoarthritis (OA) is a common degenerative joint disease characterized by progressive cartilage degradation and inflammation. In recent years, mesenchymal stem cells (MSCs) derived exosomes (MSCs-Exo) have attracted widespread attention for their potential role in modulating OA pathology. However, the unpredictable therapeutic effects of exosomes have been a significant barrier to their extensive clinical application. In this study, we investigated whether fucoidan-pretreated MSC-derived exosomes (F-MSCs-Exo) could better protect chondrocytes in osteoarthritic joints and elucidate its underlying mechanisms. In order to evaluate the role of F-MSCs-Exo in osteoarthritis, both in vitro and in vivo studies were conducted. MiRNA sequencing was employed to analyze MSCs-Exo and F-MSCs-Exo, enabling the identification of differentially expressed genes and the exploration of the underlying mechanisms behind the protective effects of F-MSCs-Exo in osteoarthritis. Compared to MSCs-Exo, F-MSCs-Exo demonstrated superior effectiveness in inhibiting inflammatory responses and extracellular matrix degradation in rat chondrocytes. Moreover, F-MSCs-Exo exhibited enhanced activation of autophagy in chondrocytes. MiRNA sequencing of both MSCs-Exo and F-MSCs-Exo revealed that miR-146b-5p emerged as a promising candidate mediator for the chondroprotective function of F-MSCs-Exo, with TRAF6 identified as its downstream target. In conclusion, our research results demonstrate that miR-146b-5p encapsulated in F-MSCs-Exo effectively inhibits TRAF6 activation, thereby suppressing inflammatory responses and extracellular matrix degradation, while promoting chondrocyte autophagy for the protection of osteoarthritic cartilage cells. Consequently, the development of a therapeutic approach combining fucoidan with MSC-derived exosomes provides a promising strategy for the clinical treatment of osteoarthritis.

**Supplementary Information:**

The online version contains supplementary material available at 10.1186/s12951-023-02264-9.

## Introduction

Osteoarthritis (OA) is a prevalent degenerative joint disease that affects millions of people worldwide [1]. It is characterized by the gradual breakdown of cartilage, chronic inflammation, and functional limitations in the affected joints [2]. It poses a significant global health burden, affecting millions of individuals and impairing their quality of life. Traditional management approaches for OA primarily focus on alleviating symptoms and improving joint function through the use of medications, physical therapy, and surgical interventions in severe cases [3, 4]. However, despite these available treatments, the burden of OA remains substantial, necessitating the exploration of innovative approaches. In recent years, there has been a growing interest in investigating novel therapeutic strategies that target the underlying mechanisms of OA, with the aim of halting or reversing disease progression. Researchers have turned their attention to emerging fields such as regenerative medicine, gene therapy, and tissue engineering, in an effort to revolutionize the approach to OA treatment [5, 6].

Regenerative medicine, particularly stem cell therapy, holds great promise in the realm of OA [7]. Mesenchymal stem cells (MSCs) possess remarkable properties, including the ability to undergo multi-lineage differentiation and self-renewal [8]. Moreover, MSCs play a crucial role in immune regulation, inflammation suppression, secretion of diverse cell growth factors, and tissue repair processes [9]. The paracrine mechanism serves as the primary mode of action for MSCs, with exosomes derived from MSCs representing a crucial avenue through which mesenchymal stem cells exert their therapeutic effects [10]. Exosomes, small extracellular vesicles released by cells, have emerged as key players in intercellular communication [11]. MSC-derived exosomes, in particular, have gained attention for their regenerative and immunomodulatory properties. They carry a diverse cargo of microRNAs (miRNAs), messenger RNAs (mRNAs), and proteins, enabling them to exert profound effects on the recipient cells through epigenetic regulation [12]. Furthermore, the contents of exosomes can dynamically change in response to environmental stimuli, altering their biological effects and enhancing their therapeutic efficacy through remodeling of the recipient cell’s epigenetic chromatin [13].

Previous studies have shown that fucoidan exhibits potent anti-inflammatory, antioxidant, anti-diabetic, and immune-modulatory effects in vitro [14–16]. It has been observed to effectively suppress M1 macrophage polarization, and demonstrate strong therapeutic efficacy in rheumatoid arthritis [15]. Additionally, research has indicated that fucoidan-loaded nanogels have the ability to reduce the release of inflammatory factors in rat chondrocytes [17]. Fucoidan and its chemical modification have aroused great interest in drug development. Therefore, we hypothesize that pretreatment of MSC-derived exosomes with fucoidan enhances their biological activity, leading to enhanced protection of osteoarthritic chondrocytes. In this study, we have developed fucoidan-pretreated exosomes derived from MSCs (F-MSCs-Exo) and demonstrated that they exhibit superior efficacy compared to MSCs-Exo in suppressing inflammatory responses and extracellular matrix degradation in osteoarthritic rats. Furthermore, F-MSCs-Exo were found to activate autophagy in the affected cells. Through further investigation, we have identified miR-146b-5p as a key component enriched in F-MSCs-Exo, which acts by silencing TRAF6 and inhibiting the PI3K/AKT/mTOR pathway. This miRNA plays a critical role in regulating both inflammation and autophagy processes.

These findings suggest that the development of exosome-based therapies utilizing a combination of fucoidan and MSCs holds great promise in providing innovative strategies for the treatment of osteoarthritis. The elucidation of the underlying mechanisms further enhances our understanding of the therapeutic potential of these approaches.

## Results

### The isolation and characterization of exosomes

**Fig. 1:**
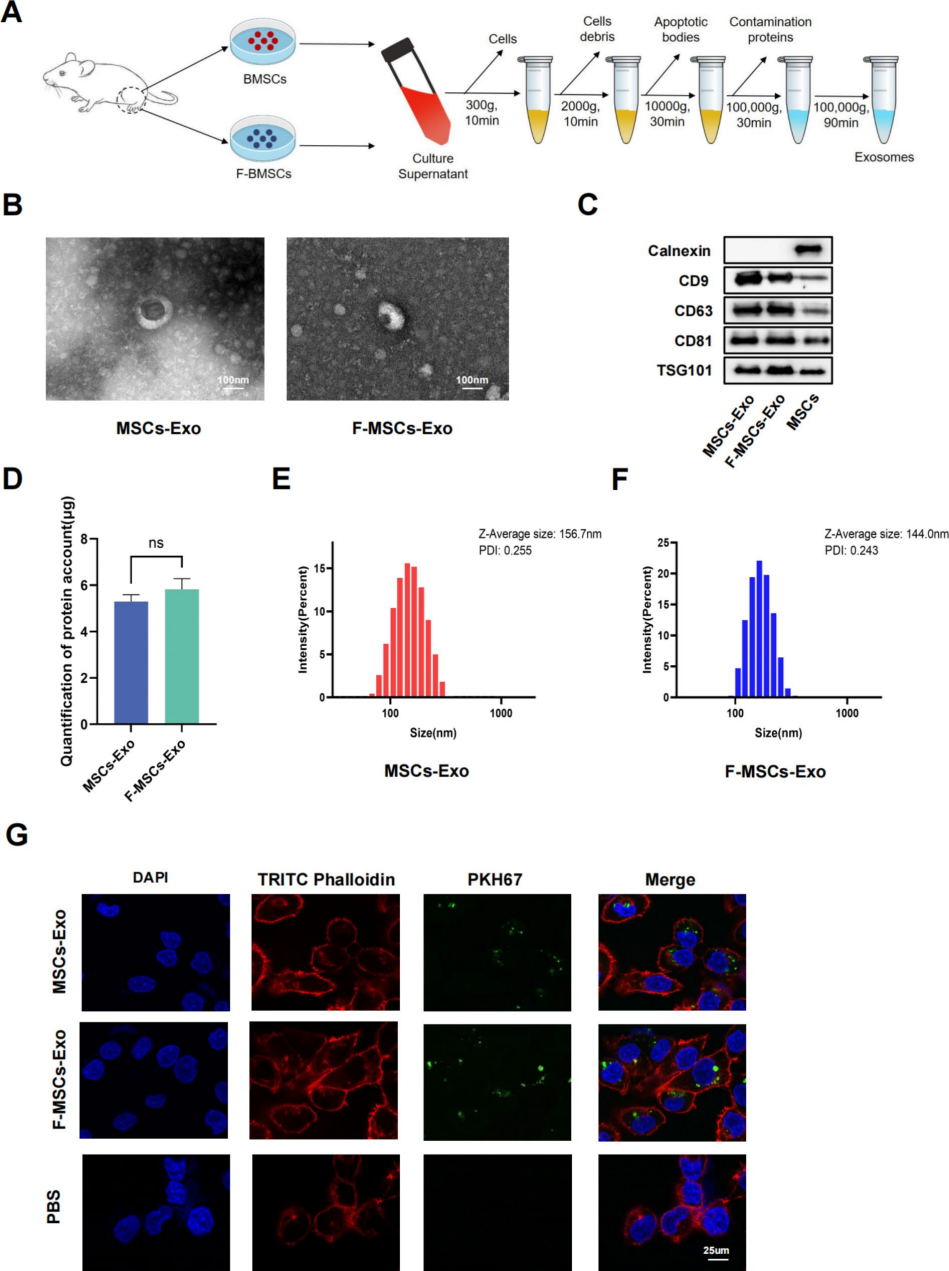
Isolation and identification of MSCs-Exo and F-MSCs-Exo. (**A**) Schematic diagram of obtaining MSCs-Exo and F-MSCs-Exo by ultracentrifugation. (**B**) TEM analysis of the morphology of MSCs-Exo and F-MSCs-Exo. (**C**) Western blot evaluation of surface markers of MSCs-Exo and F-MSCs-Exo. (**D**) Quantification of protein concentration by BCA method. (**E**, **F**) The particle size and zeta potential of MSCs-Exo and F-MSCs-Exo were analyzed by DLS method. (**G**) Cellular internalization of MSCs-Exo and F-MSCs-Exo. (ns, no significant difference; *p < 0.05; **p < 0.01; ***p < 0.001; n = 3)

Exosomes were isolated from the supernatants of bone marrow mesenchymal stem cells (MSCs) treated with or without fucoidan by ultracentrifugation (Fig. 1A). Transmission electron microscopy (TEM) was used to visualize the morphology of MSCs-Exo and F-MSCs-Exo, showing that both are membrane-intact spheres with no significant difference (Fig. 1B). The positive markers of exosomes CD9, CD63, CD81, TSG101 and the negative marker Calnexin were detected by Western blot, and there was no difference between the two (Fig. 1C). As shown in Fig. 1D, the average protein concentrations of exosomes extracted from 10^6^ cells were 5.30 ± 0.29 μg/ml (MSCs-Exo) and 5.82 ± 0.47 μg/ml (F-MSCs-Exo), respectively. Therefore, there was no difference in protein between the two. The results of dynamic light scattering (DLS) show that the average diameters of MSCs-Exo and F-MSCs-Exo are 156.7 nm and 144.0 nm, respectively, and the size ranges of both are in line with the characteristics of exosomes and there is no significant difference (Fig. 1E, F). The above results jointly confirmed the successful separation and extraction of MSCs-Exo and F-MSCs-Exo. In order to further explore the feasibility of using exosomes to treat OA, the internalization of exosomes by chondrocytes was detected, and MSCs-Exo and F-MSCs-Exo were labeled with PKH67, and the results showed that MSCs-Exo and F-MSCs-Exo can be effectively taken up by target cells (Fig. 1G). CCK-8 assay showed that treatment with the IL-1β significantly reduced the proliferation of chondrocytes, however, after MSCs-Exo and F-MSCs-Exo treatment, especially 10 μg/ml F-MSCs-Exo, significantly attenuated IL-1β-induced inhibition of chondrocyte proliferation (Fig. S1B, C).

### F-MSCs-Exo inhibit inflammatory response and M1 polarization in vitro

**Fig. 2:**
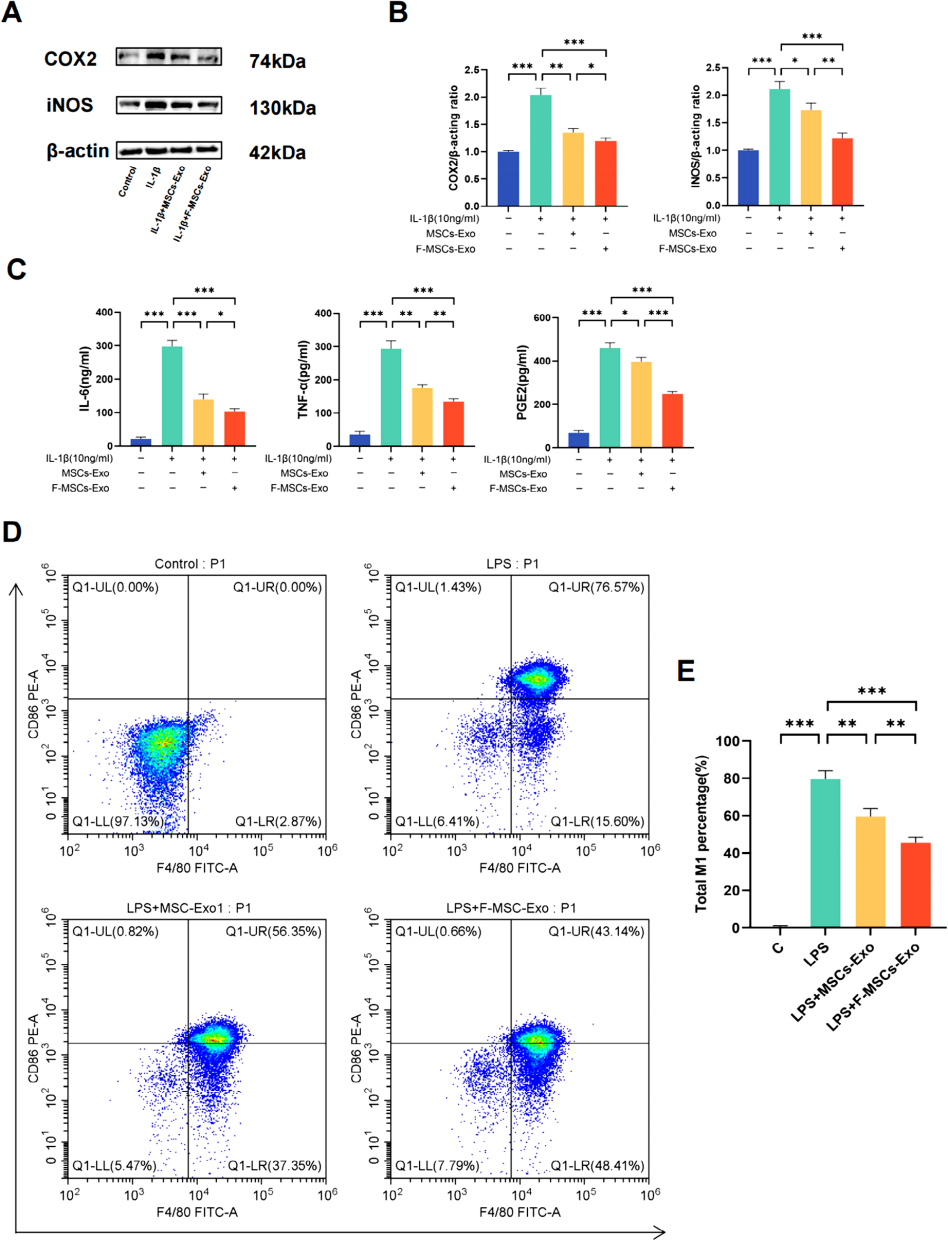
Effects of MSCs-Exo and F-MSCs-Exo on inflammatory response and M1 polarization in vitro. (**A**, **B**) Western blot analysis was performed to detect the effects of MSCs-Exo and F-MSCs-ExoOD on inflammatory factors in chondrocytes induced by IL-1β. (**C**) The levels of IL-6, TNF-α, and PGE2 in the chondrocyte culture supernatant after IL-1β induction were measured using ELISA kits to assess the impact of MSCs-Exo and F-MSCs-Exo on these inflammatory mediators. (**D**, **E**) Flow cytometry was used to investigate the influence of MSCs-Exo and F-MSCs-Exo on M1 polarization. (ns, no significant difference; *p < 0.05; **p < 0.01; ***p < 0.001; n = 3)

In recent years, a wealth of research has strongly implicated cartilage inflammation as the leading factor contributing to the development of osteoarthritis [18]. To investigate whether MSCs-Exo and F-MSCs-Exo can suppress inflammatory responses in rat chondrocytes, and whether this effect is further enhanced by pre-treatment with fucoidan, we found that after pre-treatment with 10ng/ml IL-1β, the inflammatory markers COX2 and iNOS significantly increased. However, treatment with both MSCs-Exo and F-MSCs-Exo suppressed the elevation of inflammatory markers, with F-MSCs-Exo exhibiting a stronger effect than MSCs-Exo (Fig. 2A, B). In addition, the concentrations of IL-6, TNF-α, and PGE2 in the cell supernatant were measured using ELISA assay kits, as shown in Fig. 2C, after treatment with IL-1β, these inflammatory factors increased. However, treatment with MSCs-Exo and F-MSCs-Exo reduced the release of inflammatory factors, with F-MSCs-Exo demonstrating a more significant effect. Previous studies have indicated that inflammation is primarily associated with macrophage polarization, specifically M1 macrophages, which are the main producers of pro-inflammatory cytokines [19]. In this study, Raw264.7 cells were initially treated with LPS (100ng/ml) for 24 h, followed by a 24-hour co-culture with MSCs-Exo and F-MSCs-Exo. Flow cytometry analysis revealed that M1 macrophages can be identified by their expression of CD86 and F4/80. Compared to the LPS group, both MSCs-Exo and F-MSCs-Exo significantly reduced the percentage of M1 macrophages. Notably, F-MSCs-Exo exhibited a more pronounced effect compared to MSCs-Exo (Fig. 2D, E).

### F-MSCs-Exo attenuated IL-1β-induced downregulation of anabolic markers and upregulation of catabolic markers in chondrocytes

**Fig. 3:**
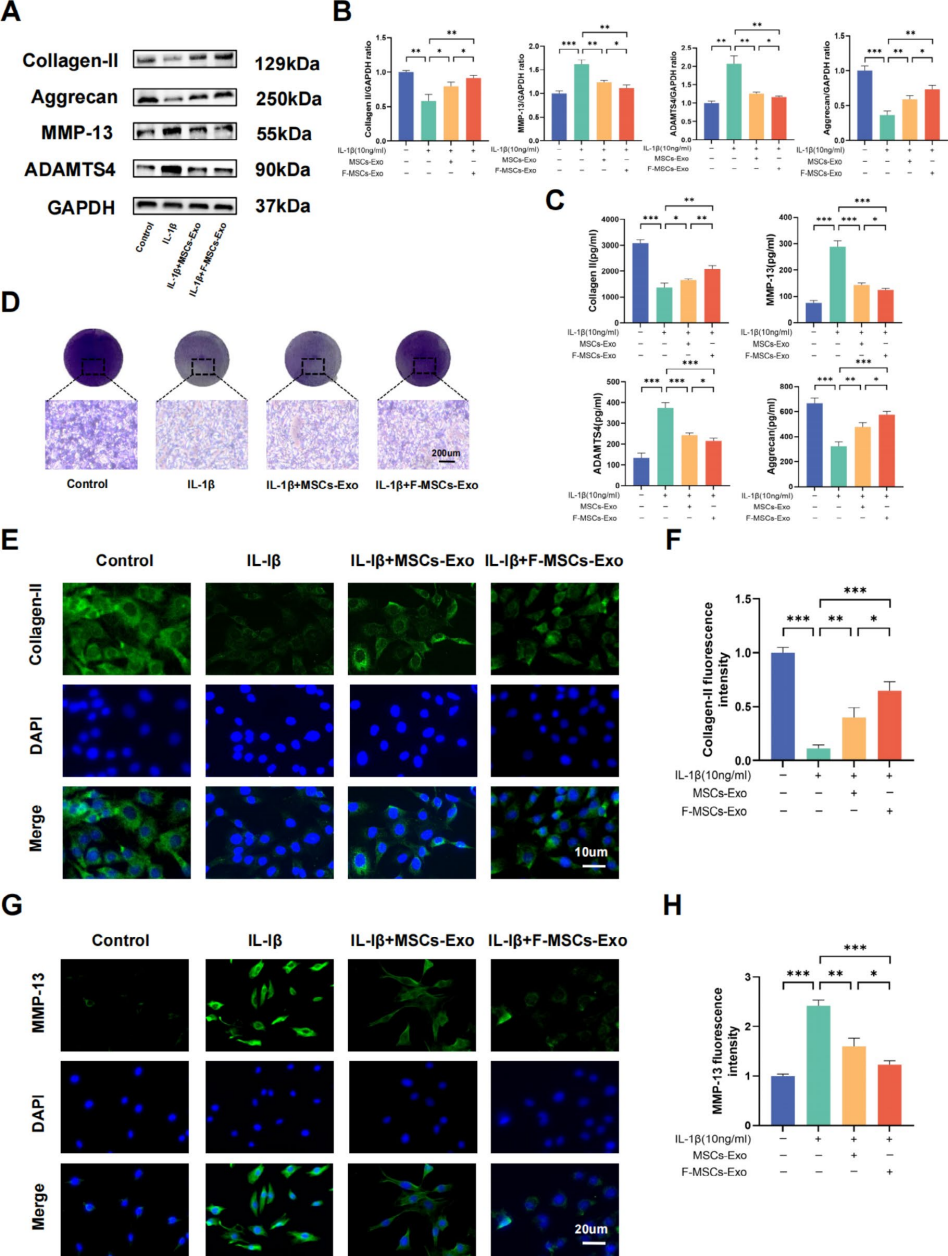
Effects of MSCs-Exo and F-MSCs-Exo on the synthesis and metabolism of cartilage extracellular matrix in vitro. (**A**, **B**) Western blot analysis was performed to detect the impact of MSCs-Exo and F-MSCs-Exo on cartilage extracellular matrix synthesis and metabolism markers. (**C**) ELISA kits were used to measure the levels of Collagen II, Aggrecan, MMP-13, and ADAMTS-4 in the cell culture supernatant. (**D**) Cartilage extracellular matrix was directly visualized using toluidine blue staining (scale bar = 200 μm). (**E** ,**F**) The expression of Collagen II was quantitatively analyzed using immunofluorescence staining and ImageJ software (scale bar = 10 μm). (**G**, **H**) The expression of MMP-13 was quantitatively analyzed using immunofluorescence staining and ImageJ software (scale bar = 20 μm). (ns, no significant difference; *p < 0.05; **p < 0.01; ***p < 0.001; n = 3)

One of the primary functions of chondrocytes is to maintain the synthesis and degradation of the extracellular matrix (ECM) [20]. To investigate the potential effects of MSCs-Exo and F-MSCs-Exo on cartilage matrix functionality, we assessed the expression of key markers involved in ECM synthesis, such as Collagen II and Aggrecan, as well as markers of degradation, including MMP-13 and ADAMTS4, in chondrocytes following IL-1β treatment. The results obtained from Western blotting and ELISA analyses demonstrated that IL-1β treatment led to a reduction in Collagen II and Aggrecan levels, while increasing the levels of MMP-13 and ADAMTS4 in rat chondrocytes. However, treatment with MSCs-Exo and F-MSCs-Exo resulted in a significant upregulation of Collagen II and Aggrecan levels, coupled with a notable downregulation of MMP-13 and ADAMTS4 levels. Importantly, F-MSCs-Exo exhibited a more pronounced effect compared to MSCs-Exo (Fig. 3A, B, C). The cartilage matrix primarily consists of cartilage proteoglycans and polysaccharides. Due to the affinity between acidic sulfate groups and basic toluidine blue dye, toluidine blue staining is commonly employed to visualize and represent the cartilage matrix [21]. Toluidine blue staining of chondrocytes also showed similar results as above (Fig. 3D). Furthermore, the results from immunofluorescence analysis also revealed a significant suppression of Collagen II downregulation and MMP-13 upregulation by F-MSCs-Exo (Fig. 3E **to H**). In summary, these results unequivocally indicate that both MSCs-Exo and F-MSCs-Exo provide protection to chondrocytes against IL-1β-induced degradation of the extracellular matrix. Furthermore, it is noteworthy that F-MSCs-Exo exhibit superior protective effects in this regard.

### F-MSCs-Exo increase IL-1β-induced autophagy in chondrocytes

**Fig. 4:**
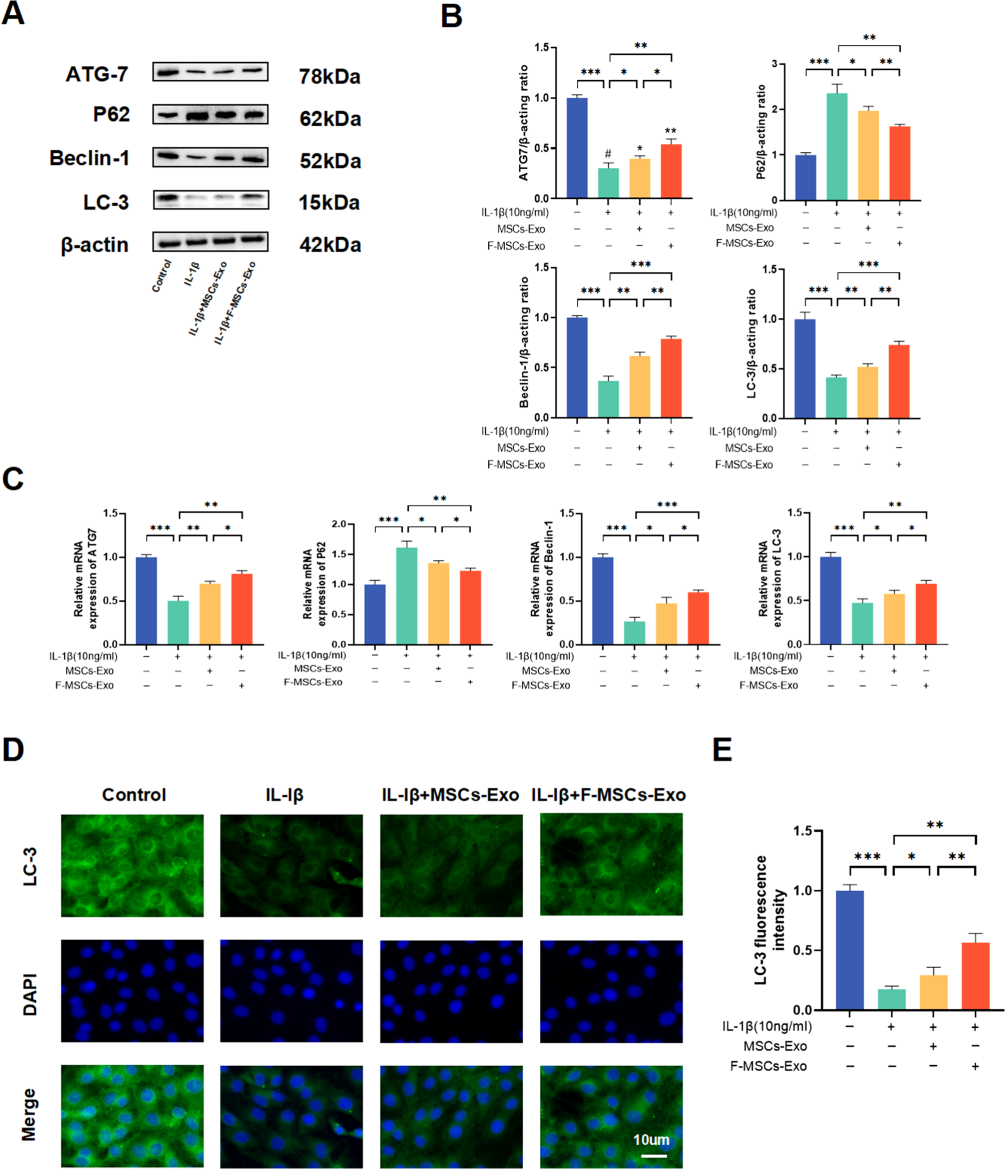
Effects of MSCs-Exo and F-MSCs-Exo on autophagy of chondrocytes in vitro. (**A**, **B**) Western blot analysis was performed to detect the impact of MSCs-Exo and F-MSCs-Exo on autophagy-related indicators of chondrocytes. (**C**) Real-time PCR technology was used to evaluate the effects of MSCs-Exo and F-MSCs-Exo on autophagy-related indicators of chondrocytes at the gene expression level. (**D**, **E**) The expression of LC-3, a marker for autophagy, was quantitatively analyzed using immunofluorescence staining and ImageJ software to provide a detailed assessment of the autophagy levels in response to MSCs-Exo and F-MSCs-Exo treatment (scale bar = 10 μm). (ns, no significant difference; *p < 0.05; **p < 0.01; ***p < 0.001; n = 3)

Emerging research suggests that inflammatory or aging chondrocytes have the ability to regulate their intracellular metabolic activity through cellular autophagy, thereby slowing down the progression of osteoarthritis [22]. The potential of F-MSCs-Exo in alleviating osteoarthritis through the modulation of chondrocyte autophagy remains a complex area of investigation. Western blotting and PCR results demonstrate that in IL-1β-stimulated chondrocytes, the expression levels of autophagy-related proteins ATG7, LC3, and beclin1 decrease, while P62 levels increase. However, treatment with both MSCs-Exo and F-MSCs-Exo activates cellular autophagy, with F-MSCs-Exo exhibiting a more remarkable effect compared to MSCs-Exo (Fig. 4A, B, C). Additionally, the results from cellular immunofluorescence similarly reveal that F-MSCs-Exo treatment enhances the expression of autophagy-related protein LC3. In other words, F-MSCs-Exo display a higher efficacy in activating cellular autophagy (Fig. 4D, E).

### Protective effect of F-MSCs-Exo on osteoarthritis in rats

**Fig. 5:**
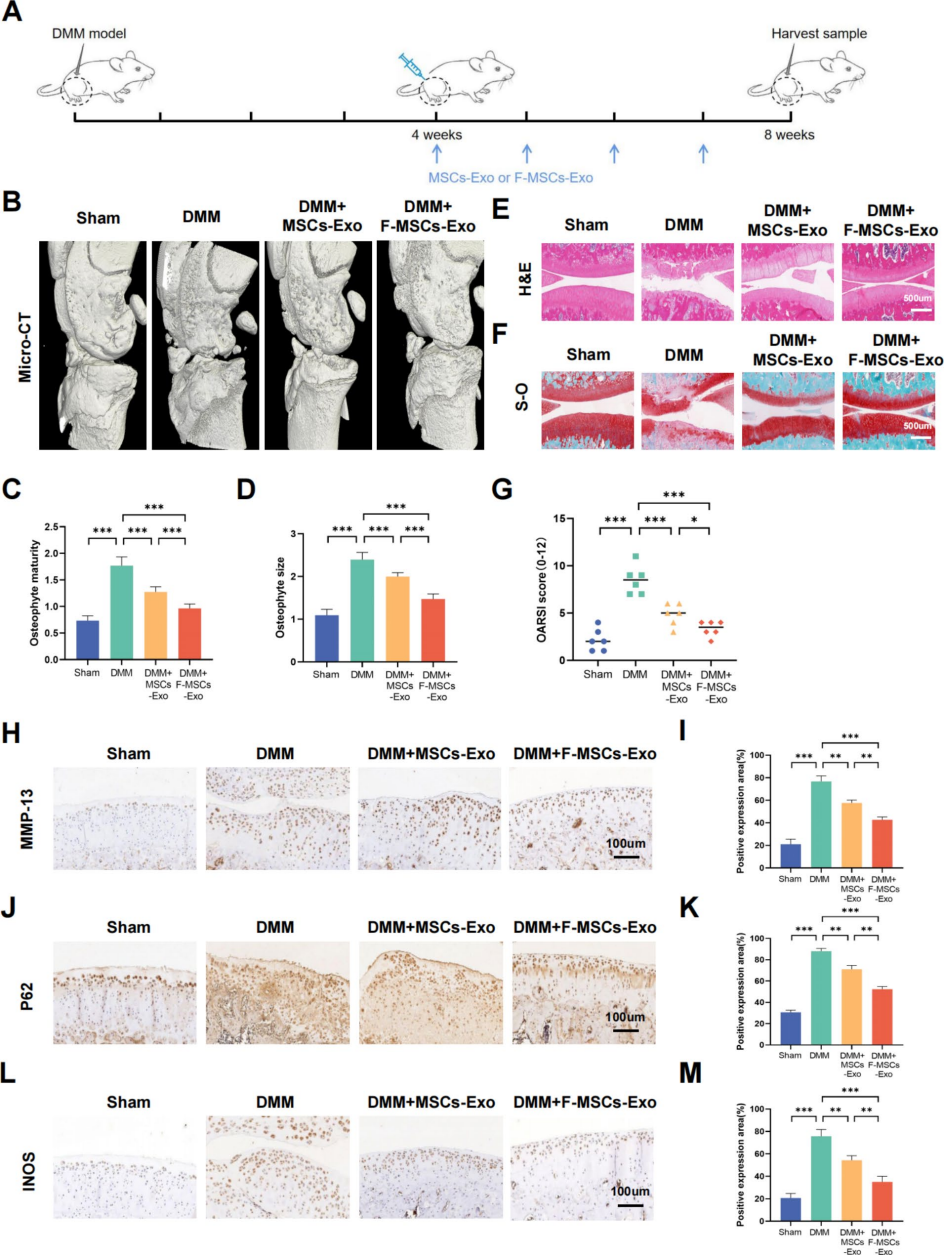
Protective effects of MSCs-Exo and F-MSCs-Exo on osteoarthritis in rats. (**A**) A schematic diagram was provided, illustrating how MSCs-Exo and F-MSCs-Exo were used to treat osteoarthritis in rats. (**B**) Micro-CT 3D reconstruction of rat knee joints was conducted to visualize the structural changes and alterations in the knee join. (**C**, **D**) Quantitative analysis of the micro-CT results was performed to assess the changes in the knee joint’s structure. (**E**, **F**) H-E staining and S-O staining of rat knee joint sections at 8 weeks after operation were conducted for histological examination (scale bar = 500 μm). (**G**) The Osteoarthritis Research Society International (OARSI) score was used to evaluate the severity of osteoarthritis in rat cartilage, n = 6. (**H** to **M**) Immunohistochemical analysis was performed to visualize and quantify the expression levels of MMP-13, P62, and INOS in the rat knee joint (scale bar = 100 μm). (ns, no significant difference; *p < 0.05; **p < 0.01; ***p < 0.001; n = 3)

To investigate the in vivo effects of MSCs-Exo and F-MSCs-Exo, a rat model of osteoarthritis was established. The animals were divided into four groups: sham surgery group, DMM (destabilization of the medial meniscus) group, DMM + MSCs-Exo group, and DMM + F-MSCs-Exo group. Briefly, as depicted in Fig. 5A, after four weeks of establishing the DMM model in rats, weekly intra-articular injections of MSCs-Exo and F-MSCs-Exo (10 μl/week) were initiated and continued for four weeks. Micro-CT is a non-invasive imaging technique that is utilized to evaluate changes in joint structure and tissues in the osteoarthritis model. The 3D reconstruction results obtained from micro-CT revealed that the DMM group of rats exhibited narrowed joint spaces, irregular joint surfaces, and an increase in osteophytes. However, following treatment with MSCs-Exo and F-MSCs-Exo, remarkable improvements were observed. The knee joint surfaces became smoother, the presence of osteophytes reduced, quantitative scores also showed that these treatments were effective, and notably, F-MSCs-Exo exhibited a significantly enhanced therapeutic effect (Fig. 5B, C, D). Furthermore, we utilized the H&E and S-O staining techniques to assess the condition of the articular cartilage tissue. In comparison to the sham-operated group, the DMM group exhibited compromised cartilage surfaces with severe erosion, as indicated by an elevated OARSI score. However, the treatment with MSCs-Exo and F-MSCs-Exo yielded remarkable improvements, resulting in smoother joint surfaces and a decrease in the OARSI score. Notably, F-MSCs-Exo exhibited a more pronounced efficacy in reducing cartilage cell erosion compared to MSCs-Exo. (Fig. 5E, F, G). Furthermore, through the implementation of immunohistochemistry and immunofluorescence techniques, we observed a significant elevation in the cartilage cell degradation marker MMP-13 and a notable decrease in the cartilage synthesis marker Collagen-II in the DMM group after 8 weeks of surgery. However, the administration of intra-articular MSCs-Exo and F-MSCs-Exo injections partially ameliorated the imbalance in extracellular matrix metabolism. Notably, treatment with F-MSCs-Exo exhibited a reduced presence of MMP-13 positive areas and an increased presence of Collagen-II positive areas, indicating a more favorable therapeutic outcome in restoring a balanced extracellular matrix metabolism profile (Fig. 5H, I and Fig. S2A, B). P62 serves as an adapter protein in autophagy, promoting the degradation of autophagy-related proteins. Interestingly, we observed a substantial upregulation of P62 expression in the DMM group. However, the administration of both MSCs-Exo and F-MSCs-Exo partially mitigated the elevation of P62. Notably, treatment with F-MSCs-Exo displayed a more pronounced inhibition of this phenomenon (Fig. 5J, K). In addition, the administration of MSCs-Exo and F-MSCs-Exo therapies has demonstrated the ability to decrease the upregulation of the inflammatory marker INOS. Moreover, treatment with F-MSCs-Exo exhibits a more pronounced reduction in the positive area associated with INOS expression (Fig. 5L, M). These findings unequivocally indicate that the therapeutic interventions using MSCs-Exo and F-MSCs-Exo effectively mitigate inflammation and extracellular matrix degradation within the rat model. Furthermore, these treatments successfully activate intracellular autophagy. Notably, F-MSCs-Exo exhibit superior efficacy, corroborating the outcomes observed in in vitro experiments.

### miR-146b-5p is a candidate effector for F-MSCs-Exo-mediated improvement in osteoarthritis

**Fig. 6:**
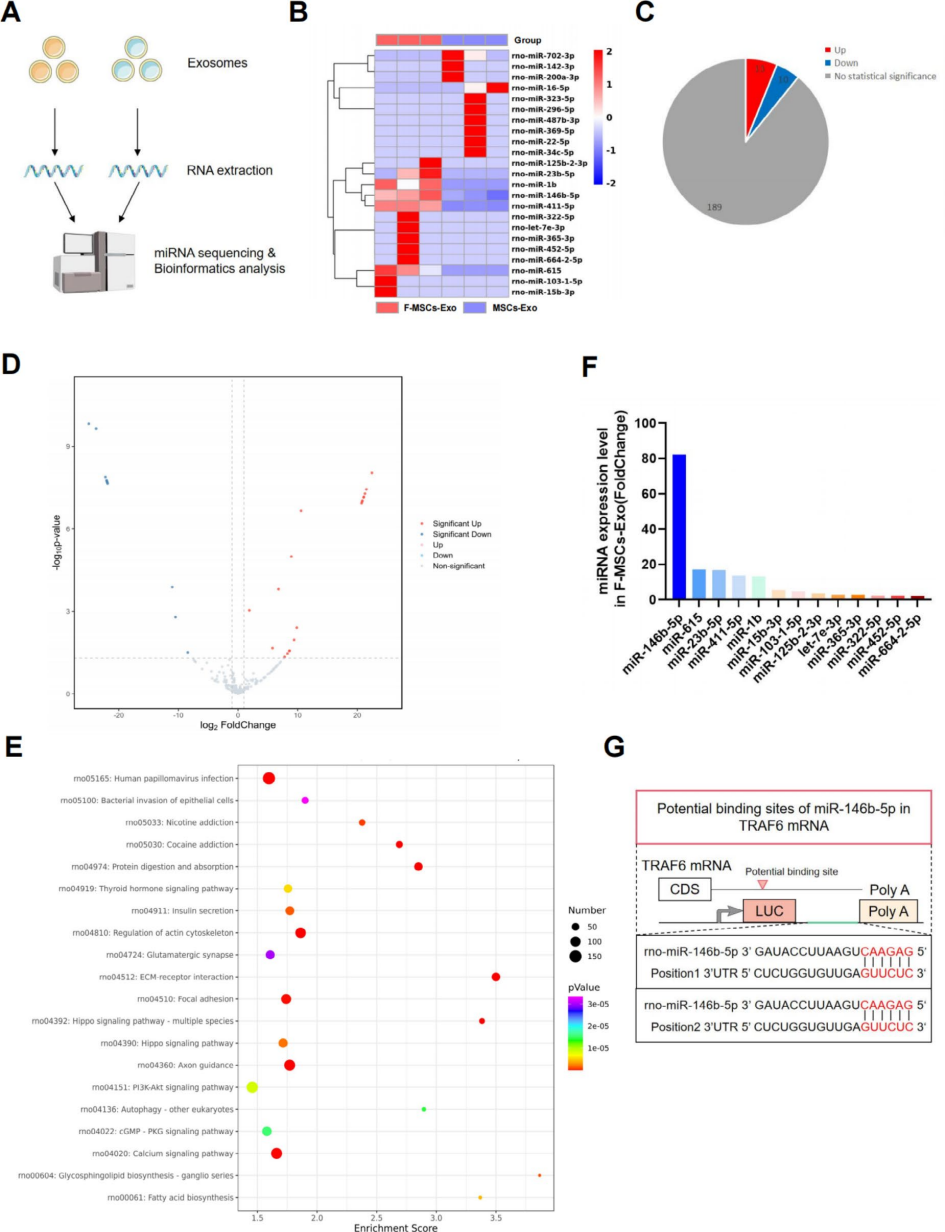
miR-146b-5p is a candidate effector for F-MSCs-Exo-mediated improvement in osteoarthritis. (**A**) A schematic diagram was presented to illustrate the process of miRNA sequencing and subsequent bioinformatics analysis. (**B**) The heat map showed the differential expression profile of miRNAs between MSCs-Exo and F-MSCs-Exo. (**C**, **D**) The pie chart and volcano plot displayed the distribution of up-regulated and down-regulated miRNAs between MSCs-Exo and F-MSCs-Exo. (**E**) KEGG enrichment analysis of F-MSCs-Exo was performed to investigate the potential biological pathways and processes affected by these miRNAs in the treatment of osteoarthritis. (**F**) Expression of up-regulated miRNAs in F-MSCs-Exo. (**G**) Targetscan was used to predict the binding site of rat miR-146b-5p and TRAF6

Numerous studies have consistently indicated that exosomes exert their biological effects by transferring specific miRNAs, which in turn regulate the functionality of target cells [11]. In order to ascertain the precise mechanisms through which F-MSCs-Exo safeguard chondrocytes in osteoarthritis, we conducted miRNA sequencing and performed bioinformatics analysis to compare the distinct miRNA expression profiles between MSCs-Exo and F-MSCs-Exo (Fig. 6A). The miRNA heatmap and volcano map analysis revealed the differential expression of miRNAs in MSCs-Exo and F-MSCs-Exo. Among the identified miRNAs, 13 exhibited significant upregulation, including rno-miR-146b-5p, rno-miR-615, rno-miR-23b-5p, rno-miR-411-5p, and rno-miR-1b. Conversely, 10 miRNAs displayed significant downregulation, such as rno-miR-142b-3p, rno-miR-200a-3p, rno-miR-702-3p, and rno-miR-16-5p. Notably, 189 miRNAs exhibited no statistically significant differences between the two groups (Fig. 6B, C, D). We performed KEGG and GO functional annotations on miRNA-related target genes. The results of the enrichment analysis revealed the significant involvement of the PI3K-AKT pathway and autophagy (Fig. 6E and Fig. S3A). Additionally, as illustrated in Fig. 6F, the most upregulated and highly expressed miRNA in F-MSCs-Exo is rno-miR-146b-5p. Considering previous research findings, there is a strong correlation between the PI3K-AKT pathway and miR-146b-5p [23]. Moreover, miR-146b-5p has been proven to play a critical role in the regulation of autophagy [24, 25]. Therefore, we have compelling reasons to believe that rno-miR-146b-5p is an effective candidate factor for the treatment of osteoarthritis using F-MSCs-Exo. Consequently, we focused our experimental efforts on further investigating rno-miR-146b-5p.

### Enriched miR-146b-5p in F-MSCs-Exo alleviates osteoarthritis by targeting TRAF6 to inhibit PI3K/AKT/mTOR pathway

**Fig. 7:**
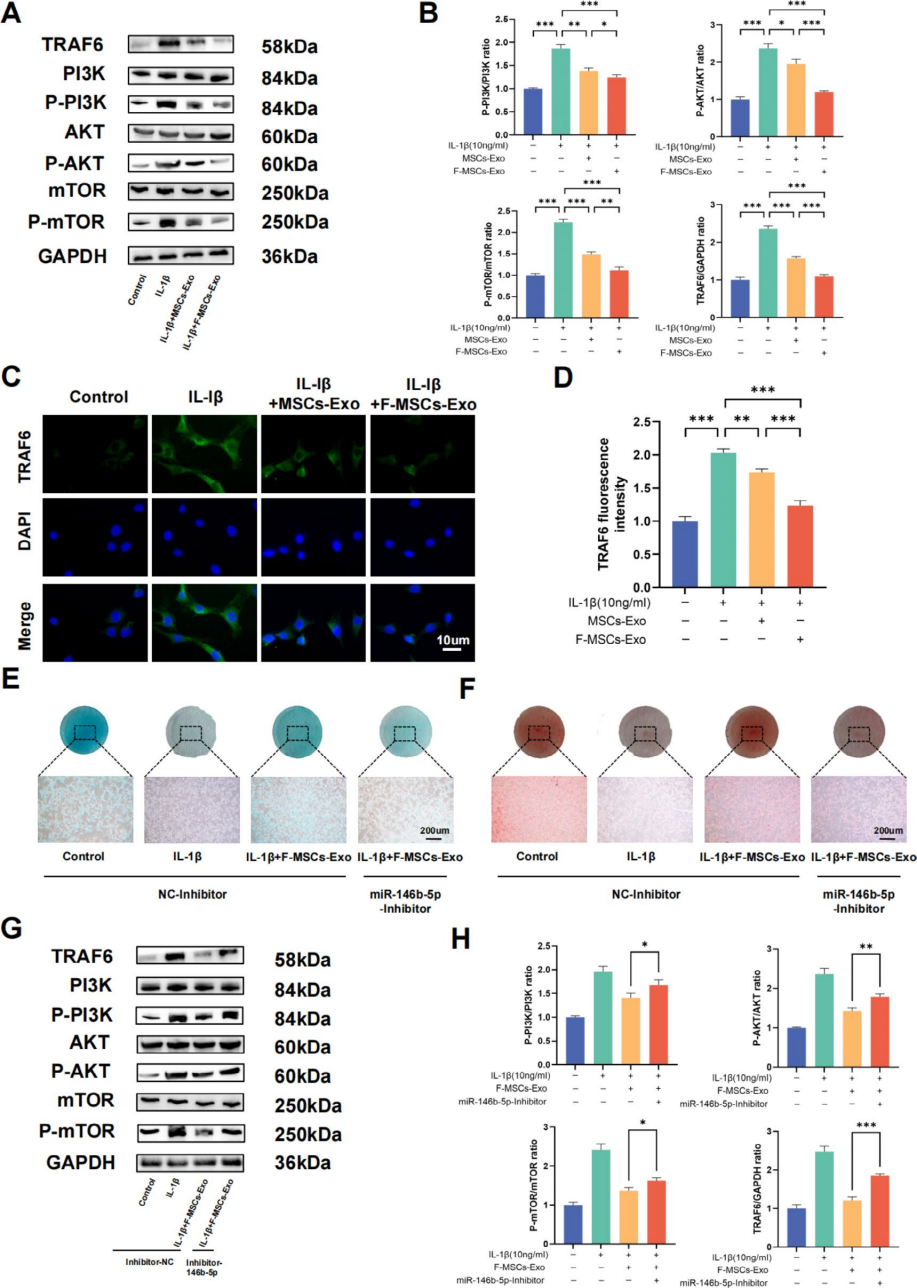
Enriched miR-146b-5p in F-MSCs-Exo inhibits PI3K/AKT/mTOR pathway by targeting TRAF6. (**A**, **B**) Western blot analysis was performed to detect the impact of F-MSCs-Exo on TRAF6 and the PI3K/AKT/mTOR pathway in rat chondrocytes. (**C**, **D**) The expression of TRAF6 was quantitatively analyzed using immunofluorescence staining and ImageJ software (scale bar = 10 μm). (**E**, **F**) Direct visualization of chondrocytes treated with nc-inhibitor and miR-146b-5p-inhibitor was performed using Alcian blue staining and safranin staining. (**G**, **H**) Western blot analysis was conducted to examine the expressions of TRAF6 and the PI3K/AKT/mTOR pathway in chondrocytes after treatment with nc-inhibitor and miR-146b-5p-inhibitor. (ns, no significant difference; *p < 0.05; **p < 0.01; ***p < 0.001; n = 3)

Numerous previous studies have consistently shown that miR-146b-5p inhibits the synthesis of inflammatory mediators by targeting tumor necrosis factor receptor-associated factor 6 (TRAF6) [26]. Additionally, the PI3K/AKT/mTOR signaling pathway plays a vital role in the normal metabolism of joint tissues. Research has demonstrated that inhibiting this pathway can alleviate osteoarthritis by promoting autophagy [27]. Notably, TRAF6 has been identified as an effective E3 ubiquitin ligase for the PI3K catalytic subunit, exerting a significant role in autophagy [28]. Overexpression of TRAF6 greatly enhances PI3K activation, resulting in AKT phosphorylation. Consequently, it is reasonable to posit a close interconnection between miR-146b-5p, TRAF6, and the PI3K/AKT/mTOR pathway. By employing protein blotting techniques, we have substantiated that both MSCs-Exo and F-MSCs-Exo can suppress the elevated expression of TRAF6 and the PI3K/AKT/mTOR pathway induced by IL-1β. Importantly, F-MSCs-Exo exhibited more pronounced effects compared to MSCs-Exo (Fig. 7A, B). The results from cellular immunofluorescence also support the superior inhibitory potential of F-MSCs-Exo on the IL-1β-induced upregulation of TRAF6 (Fig. 7C, D).

To further explore whether F-MSCs-Exo can exert its inhibitory effects on the PI3K/AKT/mTOR pathway by silencing TRAF6 *via* miR-146b-5p, we conducted an analysis using the Targetscan database [29]. This analysis revealed the existence of a specific binding region between miR-146b-5p and TRAF6 (Fig. 6G). Additionally, our hypothesis was reinforced by the use of a miR-146b-5p inhibitor. Staining results with Alizarin blue and Fast Red demonstrated that the therapeutic effects of F-MSCs-Exo were inhibited by the miR-146b-5p inhibitor (Fig. 7E, F). Furthermore, protein blotting results indicated that F-MSCs-Exo effectively suppressed the IL-1β-induced upregulation of TRAF6 and the PI3K/AKT/mTOR pathway, whereas the miR-146b-5p inhibitor attenuated this effect (Fig. 7G, H). These findings strongly suggest that miR-146b-5p, enriched in F-MSCs-Exo, exerts its inhibitory effects on the PI3K/AKT/mTOR pathway by targeting TRAF6.

### Antagomir-146b-5p reversed the protective effect of F-MSCs-Exo on osteoarthritis in rats

**Fig. 8:**
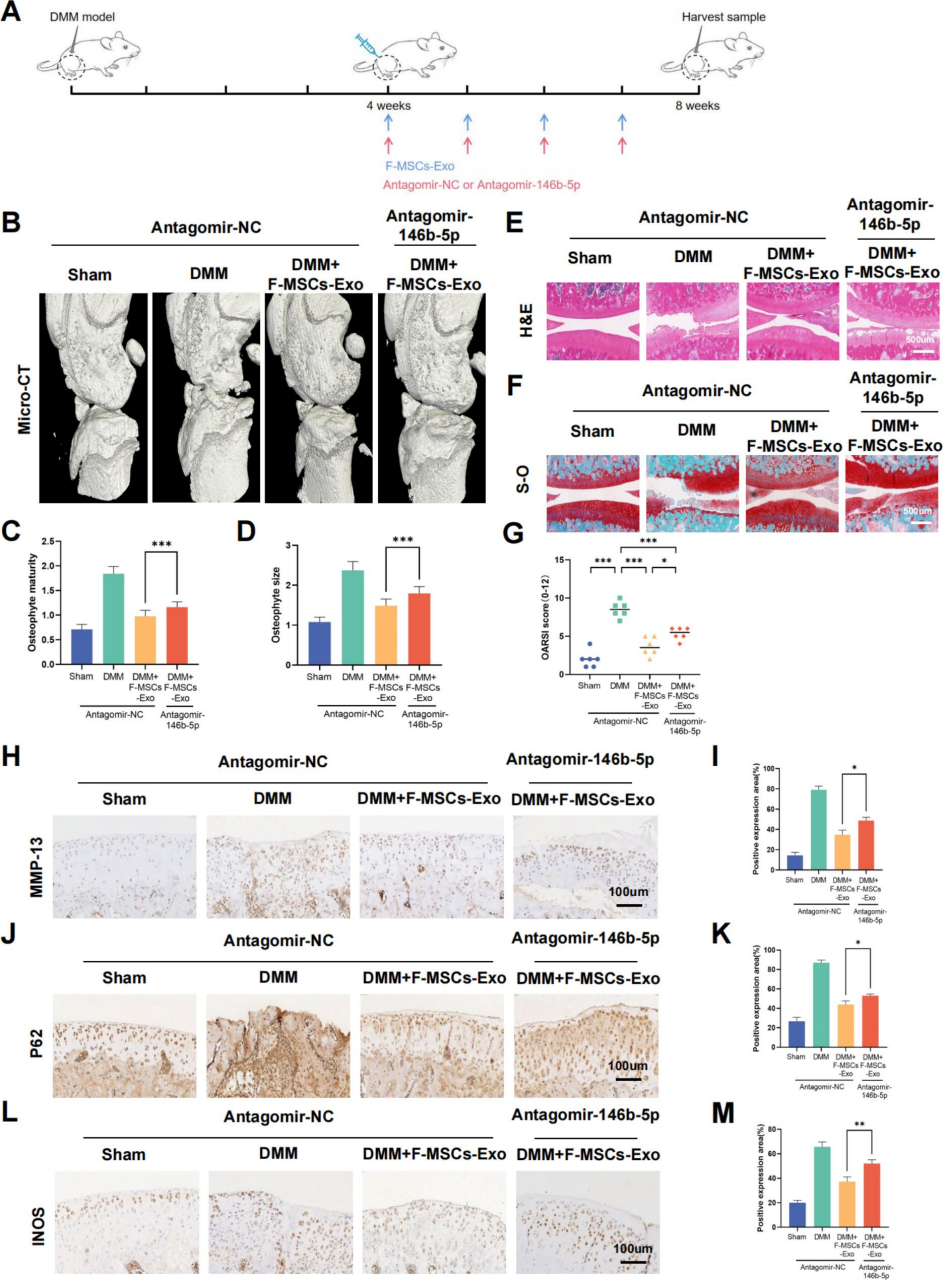
Antagomir-146b-5p reverses the therapeutic effect of F-MSCs-Exo on osteoarthritis in rats. (**A**) Schematic diagram of the experiment evaluating whether miR-146b-5p is involved in the treatment of osteoarthritis in rats by F-MSCs-Exo. (**B**) Micro-CT 3D reconstruction of the rat knee joint was performed to visualize structural changes and alterations caused by Antagomir-146b-5p treatment. (**C**, **D**) Quantitative analysis of micro-CT results. (**E**, **F**) H-E staining and S-O staining of rat knee joint sections were conducted for histological examination, enabling the assessment of tissue morphology and cartilage integrity (scale bar = 500 μm). (**G**) The Osteoarthritis Research Society International (OARSI) score was used to evaluate the severity of osteoarthritis in rat cartilage, n = 6. (**H** to **M**) Immunohistochemical analysis was performed to visualize and quantify the expression levels of MMP-13, P62, and INOS in the rat knee joint after Antagomir-146b-5p treatment (scale bar = 100 μm). (ns, no significant difference; *p < 0.05; **p < 0.01; ***p < 0.001; n = 3)

To investigate the potential involvement of miR-146b-5p in the therapeutic efficacy of F-MSCs-Exo for treating osteoarthritis in rats, we utilized Antagomir-146b-5p, a modified miRNA antagonist. In brief, as depicted in Fig. 8A, we established a rat model of destabilization of the medial meniscus (DMM) and, after a 4-week period, initiated weekly intra-articular injections of F-MSCs-Exo (10 μl/week) for a duration of 4 weeks. Concurrently, starting from the 4th week, we administered Antagomir-NC (5nmol/week) or Antagomir-146b-5p (5nmol/week) *via* intra-articular injection using a microliter syringe. The results of 3D knee joint reconstruction using Micro-CT reveal that the articular surface of the F-MSCs-Exo + Antagomir-146b-5p group appears less smooth compared to the F-MSCs-Exo + Antagomir-NC group. Additionally, there is an increase in the formation of osteophytes, resulting in a significantly higher osteophyte score in the F-MSCs-Exo + Antagomir-146b-5p group (Fig. 8B, C, D). H&E and S-O staining results also demonstrate that the use of Antagomir-146b-5p eliminates the therapeutic effects of F-MSCs-Exo on osteoarthritis, leading to a significant increase in the OARIS score (Fig. 8E, F, G). Furthermore, immunohistochemistry analysis shows that F-MSCs-Exo effectively reduces the positive areas of MMP-13, iNOS, and P62. However, the use of Antagomir-146b-5p significantly diminishes the inhibitory effects of F-MSCs-Exo on MMP-13, iNOS, and P62 (Fig. 8H to M). In summary, the findings suggest that Antagomir-146b-5p can partially reverse the extracellular matrix degradation, inflammation inhibition, and activation of autophagy mediated by F-MSCs-Exo. This further supports the notion that F-MSCs-Exo can deliver miR-146b-5p to the rat knee joint, silence TRAF6, and subsequently inhibit the PI3K/AKT/mTOR pathway to protect chondrocytes in osteoarthritis.

## Discussion

Osteoarthritis is currently the most prevalent chronic joint disease, characterized by degenerative changes in cartilage and surrounding tissues [30]. The primary destructive forces in this condition are cartilage degradation and inflammation [31]. Current treatments for osteoarthritis primarily focus on symptom relief and improving quality of life, often relying on medications such as non-steroidal anti-inflammatory drugs and pain relievers [32]. While these approaches can alleviate pain and improve joint function, they do not reverse or halt the progression of the disease or restore damaged joint tissues. Therefore, there is a need to explore more innovative and effective treatment methods to achieve long-term disease control and potential cure.

In recent years, new treatment strategies have garnered attention, with mesenchymal stem cell-derived exosomes (MSCs-Exo) emerging as a promising approach for osteoarthritis therapy [33]. MSCs-Exo are vesicular structures released by MSCs, containing a diverse range of bioactive molecules that can improve disease processes by modulating cellular signaling pathways and promoting tissue regeneration [34]. The proteins, nucleic acids, and growth factors within these vesicles play a crucial role in treatment outcomes. Research has demonstrated that MSCs-Exo contain abundant anti-inflammatory factors and immune regulatory factors, which can alleviate symptoms of osteoarthritis by suppressing inflammatory reactions, reducing inflammatory cell infiltration, and modulating immune cell functions [35, 36]. Studies by researchers such as Zhang have shown that MSCs-Exo have the ability to promote the proliferation and differentiation of chondrocytes, stimulating the regeneration and repair processes of cartilage cells [37]. Additionally, Liu found that MSCs-Exo can inhibit enzymes associated with cartilage degradation and reduce chondrocyte apoptosis [38]. Overall, MSCs-Exo hold great promise in the treatment of osteoarthritis, but further research is needed to fully understand their biological mechanisms and therapeutic effects. Recent reports have highlighted the enhanced functionality of MSCs-Exos through exogenous preconditioning. Zhang et al. found that extracellular vesicles subjected to hypoxic preconditioning significantly enhanced chondrocyte proliferation and inhibited chondrocyte apoptosis [39]. Shao et al. demonstrated that exosomes from infrapatellar fat pad-derived MSCs pre-treated with Kartogenin enhanced cartilage regeneration and chondrocyte synthetic metabolism [40]. Thus, it is evident that exogenous preconditioning can influence the functionality of MSCs-Exo.

Fucoidan, a naturally occurring polysaccharide compound extracted from brown algae, has been extensively studied due to its intriguing biological activities [41]. Particularly, its anti-diabetic and anti-cancer properties have received significant research attention over the past decade [42, 43]. In recent years, the anti-inflammatory properties of fucoidan have been explored, and its application in the treatment of various diseases has been investigated. He et al. found that fucoidan could inhibit M1 polarization of macrophages, thereby improving colitis [44]. Liu et al. demonstrated that fucoidan treated ulcerative colitis by modulating gut microbiota and bile acid metabolism [45]. Phull AR et al.‘s research suggested that fucoidan effectively alleviated oxidative stress, thus providing a potential treatment for rheumatoid arthritis [46]. Based on these experiments, we selected fucoidan as a pre-treatment agent due to its potent anti-inflammatory effects. It is hypothesized that Fucoidan-Modified MSCs-Exosomes (F-MSCs-Exo) will exhibit enhanced therapeutic effects for osteoarthritis.

**Fig. 9:**
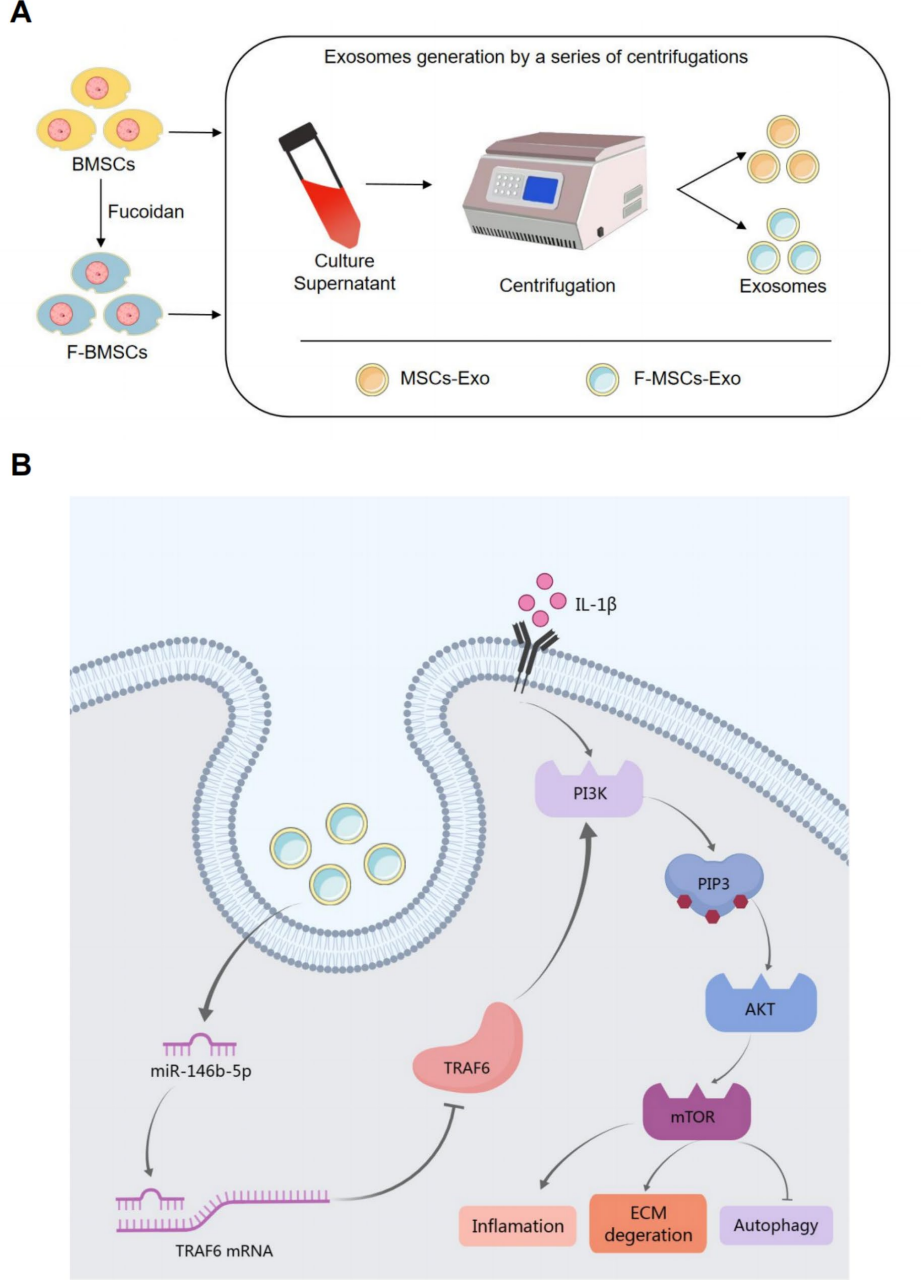
Schematic diagram of the protective effect of F-MSCs-Exo on OA. (**A**) Obtain MSCs-Exo and F-MSCs-Exo by ultracentrifugation. (**B**) Enriched miR-146b-5p in F-MSCs-Exo plays a role in the treatment of osteoarthritis by targeting TRAF6 and inhibiting PI3K/AKT/mTOR signaling pathway

In our research, we prepared MSCs-Exos and F-MSCs-Exos after pretreating them with fucoidan (Fig. 9A). We characterized the exosomes using various techniques such as TEM, DLS, and BCA. The results showed no significant statistical differences in morphology, particle size, yield, and protein concentration between MSCs-Exos and F-MSCs-Exos. Additionally, both types of exosomes were effectively internalized by chondrocytes. In our in vitro experiments, we observed that F-MSCs-Exos significantly reduced the expression of inflammation in chondrocytes induced by IL-1β, compared to MSCs-Exos. We also found that this reduction in inflammation was associated with a decrease in M1 polarization of macrophages. Moreover, F-MSCs-Exos demonstrated superior abilities in maintaining the extracellular matrix and activating autophagy, surpassing the performance of MSCs-Exos. In our in vivo experiments, we used micro-CT3D reconstruction, H&E staining, S-O staining, and immunohistochemistry to evaluate the therapeutic efficacy of F-MSCs-Exos for treating osteoarthritis. The results consistently supported the superior treatment capacity of F-MSCs-Exos compared to MSCs-Exos.

To explain the underlying mechanism behind the enhanced bioactivity of MSCs-Exos after fucoidan pretreatment, we analyzed the differential expression profiles of miRNAs between MSCs-Exos and F-MSCs-Exos. The analysis revealed significant upregulation of miR-146b-5p, miR-615, miR-23b-5p, miR-411-5p, and miR-1b in F-MSCs-Exos, with miR-146b-5p exhibiting the highest expression level. KEGG enrichment analysis indicated the involvement of the PI3K-AKT pathway and autophagy in the functionality of F-MSCs-Exos. The PI3K/AKT pathway, a regulator of autophagy, plays a crucial role in chondrocyte metabolism. Prior studies have shown that artesunate modulates cellular autophagy through the PI3K/AKT/mTOR signaling pathway [47]. Through the Targetscan database, we identified a specific relationship between TRAF6 and miR-146b-5p. It has been demonstrated that miR-146b-5p inhibits the synthesis of inflammatory mediators by targeting tumor necrosis factor receptor-associated factor 6 (TRAF6), which is a key player in autophagy induction [26].

Based on these findings, we propose that the enriched miR-146b-5p in F-MSCs-Exos directly targets TRAF6, thereby inhibiting the PI3K/AKT/mTOR signaling pathway (Fig. 9B). This mechanism contributes to the anti-inflammatory effects, maintenance of the extracellular matrix, and activation of autophagy observed in chondrocytes. In vitro experiments confirmed that F-MSCs-Exos treatment effectively suppressed the activation of TRAF6 and the PI3K/AKT/mTOR signaling pathway, surpassing the effects of MSCs-Exos. We further demonstrated the involvement of miR-146b-5p by using a miR-146b-5p inhibitor, which partially reversed the inhibitory effects of F-MSCs-Exos on TRAF6 and the PI3K/AKT/mTOR signaling pathway. Moreover, in vivo experiments using an antagonist, Antagomir-146b-5p, injected into the joint cavity of rats, also partially reversed the therapeutic effects of F-MSCs-Exos on osteoarthritis. Overall, our study provides strong evidence supporting the direct targeting of TRAF6 by miR-146b-5p enriched in F-MSCs-Exos, leading to the inhibition of the PI3K/AKT/mTOR signaling pathway. This process contributes to the suppression of inflammation, degradation of the extracellular matrix, and activation of autophagy in chondrocytes.

Due to the enhanced paracrine effects achieved through MSC pre-processing, F-MSCs-Exo not only manifest the aforementioned benefits but also guard against enzymatic degradation in bodily fluids. Furthermore, exosome transplantation presents advantages such as non-tumorigenicity, non-immunogenicity, and ease of storage and transport. However, the safety and efficacy of F-MSCs-Exo therapy still necessitate further clinical trials for validation. Additionally, although there is evidence indicating that TRAF6 may act as a downstream target protein for miR-146b-5p within F-MSCs-Exos, it is evident that other miRNAs within exosomes can also impact TRAF6 and other proteins. For instance, miR-23b-5p targets TRAF6 to inhibit cell apoptosis and alleviate myocardial inflammation [48], while miR-615 can suppress the PI3K-AKT pathway, influencing the apoptosis of hippocampal neurons [49]. Therefore, it is crucial to consider the potential synergistic effects of these miRNAs, and studying miR-146b-5p and TRAF6 in isolation may oversimplify their true biological characteristics.

## Conclusion

In conclusion, pretreatment with fucoidan significantly enhances the efficacy of MSC-derived exosomes in suppressing cartilage inflammation, degrading the extracellular matrix, and activating autophagy. It also demonstrates a more effective protective role in a rat model of osteoarthritis. Additionally, our study further elucidates that F-MSCs-Exo exerts its effects by targeting TRAF6 through the enrichment of miR-146b-5p, consequently inhibiting the PI3K/AKT/mTOR pathway. This research introduces a novel approach for the endogenous modification of MSC-derived exosomes using fucoidan and sheds light on its potential regulatory mechanism. In essence, F-MSCs-Exo emerges as a satisfying candidate drug for the treatment of osteoarthritis and holds promise for clinical application. This paves the way for a prospective and innovative therapeutic approach to clinical osteoarthritis treatment, suggesting that a future non-cellular therapeutic strategy could involve the combination of exosomes with miRNA.

## Materials and methods

### Cell culture

Mesenchymal stem cells (MSCs) were isolated and cultured from bone marrow using a previously established method. In simple terms, bone marrow stromal cells (BMSCs) were isolated from the femurs of 4-week-old male Sprague-Dawley rats (90–100 g) and then cultured in α-MEM (Minimum Essential Medium) supplemented with 1% penicillin-streptomycin (Gibco, NY, USA) and 10% fetal bovine serum (FBS, Gibco, USA) [50]. The cells were incubated at 37 °C in a 5% CO2 environment. Passage 3–4 BMSCs were selected for subsequent experiments. Chondrocytes were obtained from rat knee joints. The small pieces of cartilage tissue (roughly 1mm^3^ in size) were subjected to digestion using 0.2% type II collagenase ( Sigma-Aldrich, USA) at a temperature of 37 °C for a duration of 6 h [51]. Following centrifugation at 1500 rpm for 5 min, the cells were suspended again in DMEM/F-12 and placed in culture within DMEM/F-12 medium. This medium was enriched with 1% penicillin-streptomycin and 10% fetal bovine serum, all under identical conditions as those for BMSCs. Passage 1–3 chondrocytes were used for cellular experiments. Raw264.7 cells (Beyotime, Shanghai) were cultured in DMEM (Dulbecco’s Modified Eagle Medium) containing 10% FBS and 1% penicillin-streptomycin [52]. For the treatment of BMSCs, fucoidan (Santa Cruz, USA) was used for pretreatment (Fig. S1A) [53]. After 48 h of pretreatment, the BMSCs were collected for exosome extraction.

### MSCs-Exo and F-MSCs-Exo preparation and characterization

MSCs-derived exosomes (MSCs-Exo) and Fucoidan-preconditioned MSCs-derived exosomes (F-MSCs-Exo) were isolated from the supernatant using an ultracentrifugation-based method [54]. In short, the supernatant was collected and centrifuged at 300 and 2000 g for 10 min each, respectively, to remove dead cells and cell debris. Subsequently, an additional centrifugation step at 10,000 g for 30 min was performed to eliminate apoptotic bodies. Afterward, ultracentrifugation at 100,000 g for 30 min using Ultraclear™ tubes (Beckman Coulter, USA) was carried out to remove residual proteins. Finally, the purified samples were resuspended in PBS following centrifugation at 100,000 g for 90 min and stored at -80 °C for further experiments. The total protein concentration in the exosomes was determined using the bicinchoninic acid assay (BCA) protein detection kit (Beyotime, China). The size distribution and zeta potential of the exosomes were measured using a Zetasizer Nano ZS90 (Malvern, UK) through dynamic light scattering (DLS). Furthermore, the morphology of the exosomes was examined using transmission electron microscopy (TEM, Hitachi, Japan). Additionally, Western blotting was employed to identify the expression of the marker proteins CD9, CD63, CD81, TSG101, and Calnexin (ProteinTech, USA) in the exosomes.

### Cellular uptake assay

Exosomes were labeled using the green fluorescent dye PKH67 (Sigma-Aldrich, USA), while the cellular cytoskeleton was labeled with the red fluorescent dye TRITC Phalloidin (Thermo Fisher Scientific, USA). The co-culture of exosomes (10 μg/ml) with rat chondrocytes took place in a serum-free medium at 5% CO2 and 37 °C for 12 h. Subsequently, the cells were fixed with 4% paraformaldehyde. DAPI (Beyotime, China) was used for nuclear staining. Finally, the uptake of exosomes was visualized and captured using a fluorescence microscope (Zeiss, Germany).

### Cell viability assay

The effects of MSCs-Exo and F-MSCs-Exo on cell viability were analyzed using the CCK-8 method. Rat chondrocytes (8*10^3^ cells per well) were seeded into the wells of a 96-well plate and allowed to incubate for 12 h. Subsequently, the cells were treated with varying concentrations (0, 1, 5, 10 μg/ml) of MSCs-Exo, as well as with 10 μg/ml of both MSCs-Exo and F-MSCs-Exo [39]. After 24, 48, and 72 h of treatment, 10 μl of CCK-8 solution (Meilunbio, China) was added to each well, followed by a 4-hour incubation at 37 °C [55]. The absorbance was measured at 450 nm using a microplate reader (Leica Microsystems, Germany) to assess cell viability.

### Cell toluidine blue, alcian blue, safranin O staining

Rat chondrocytes were seeded into a 24-well plate and allowed to grow until they reached 75% confluency. Afterward, the cells were gently washed three times with PBS and fixed with 4% paraformaldehyde for 15 min, followed by another round of PBS washing. Subsequently, carefully add the respective staining solutions, including toluidine blue, Alcian blue, and safranin O (solarbio, China), to the wells, and let them incubate undisturbed at room temperature for 30 min. Finally, remove the excess dye by washing with PBS, and observe the stained cells under an ordinary light microscope.

### Western blotting

For protein extraction, chondrocytes from the 1st to 3rd passages were utilized. In brief, the cells were lysed in RIPA lysis buffer (Beyotime, China) containing protease and phosphatase inhibitors. The lysate was maintained on ice for 15 min, after which it underwent centrifugation at 12,000 rpm for 30 min at a temperature of 4 °C. This procedure was performed to gather the resultant supernatant. Using the BCA method, the protein concentration was established. Following this, 15 μg of protein was segregated on an 8–12% SDS-PAGE gel and subsequently transferred to a polyvinylidene fluoride (PVDF) membrane. The membrane was blocked with 5% non-fat milk at room temperature for 2 h and then incubated overnight at 4 °C with the primary antibody. After washing, the membrane was incubated with an HRP-conjugated secondary antibody at 37 °C for 2 h. Finally, protein imaging and quantification were performed using the ChemiDoc™ XRS + and Image Lab 3.0 imaging system (Bio-Rad, USA).

The experiment utilized the following primary antibodies: COX2 (27308-1-AP), INOS (18985-1-AP), Collagen II (28459-1-AP), Aggrecan (13880-1-AP), MMP-13 (18165-1-AP), ADAMTS4 (11865-1-AP), all of which were purchased from Proteintech Group. Additionally, the antibodies ATG7 (ab133528), LC3 (ab192890), beclin1 (ab207612), P62 (ab109012), and TRAF6 (ab40675) were acquired from Abcam. The antibodies PI3K (AF6241), AKT (AF0836), mTOR (AF6308), P-PI3K (AF3242), P-AKT (AF0016), P-mTOR (AF3308), GAPDH (AF7021), and β-actin (AF7018) were purchased from Affinity Biosciences. Goat anti-rabbit and anti-mouse IgG-HRP antibodies were also procured from Affinity Biosciences.

### Enzyme-linked immunosorbent assay (ELISA)

The cell culture supernatant is collected and stored at -20 °C for subsequent ELISA testing. In each well, the concentrations of IL-6, TNF-α, Collagen II, Aggrecan, MMP-13, and ADAMTS4 were quantified using ELISA kits [56]. Concisly, the cell culture supernatant was introduced onto a plate, followed by the introduction of a primary antibody to establish binding with the antigen present in the sample. Subsequently, an enzyme-labeled secondary antibody was administered onto the plate, leading to the formation of a complex. Finally, a substrate was added to initiate the enzyme-catalyzed color reaction. The absorbance was measured using a spectrophotometer (Leica Microsystems, Germany) to calculate the specific protein or molecule’s concentration.

### Real-time quantitative reverse transcription PCR (RT-qPCR)

Total mRNA was extracted from rat chondrocytes using TRIzol reagent (Sangon, China), and the RNA content was quantified using the Nanodrop 2000 spectrophotometer. cDNA synthesis was performed using the cDNA synthesis kit (Takara, Japan). RT-qPCR was conducted with the SYBR Green detection reagent (Takara, Japan) on the LightCycler® 96 real-time PCR system (Roche, USA). The relative expression levels of mRNA were determined using the 2 − ΔΔCq method. The primer sequences for mRNA can be found in Table S1.

### Immunofluorescence

In brief, the cells were subjected to a series of steps as follows: first, they were washed with PBS and then fixed with 4% formaldehyde for 15 min. After another round of washing, the cells were permeabilized using 0.2% Triton X-100 for 15 min at room temperature. Subsequently, the cells were treated with 10% goat serum for 30 min at 37 °C to block non-specific binding. Next, the cells were incubated overnight with a panel of primary antibodies, namely Collagen II (1:200), MMP-13 (1:200), LC3 (1:200), and TRAF6 (1:200). On the following day, the cells were exposed to secondary antibodies conjugated with Alexa®488 (1:400) for 1 h. Following another wash, the samples were stained with 4’,6-diamidino-2-phenylindole (DAPI) for 60 s to visualize the cell nuclei [57]. Finally, the samples were visualized under a fluorescence microscope (Carl Zeiss, Germany), and the ImageJ software was employed for quantitative analysis in each specified region.

### Flow cytometry analysis

Raw264.7 cells were cultured in a 6-well plate until they reached 80-90% confluency. Upon reaching the desired confluency, the cells were carefully harvested and collected in centrifuge tubes after being washed with PBS. To minimize non-specific antigen binding, the cells were then incubated with 3% BSA for 1 h. After centrifugation, the cells were resuspended in PBS and incubated at 37 °C in a light-protected environment along with the following fluorescently-labeled antibodies: FITC-conjugated anti-F4/80 antibody and PE-conjugated anti-CD86 antibody (BD Biosciences, USA) for 30 min [58]. Subsequently, a thorough washing step was performed to remove any unbound antibodies, and the samples were subjected to flow cytometry for analysis.

### miRNA sequencing

Total RNA was extracted using the mirVana miRNA Isolation Kit (Ambion). The RNA quantity was assessed with Nanodrop 2000 (Thermo Fisher Scientific Inc., USA), while its integrity was evaluated using the Agilent 2100 Bioanalyzer (Agilent Technology, USA). To construct small RNA libraries, the NEBNext Small RNA Library Prep Set for Illumina kit (NEB, USA) was utilized. After confirming the high-quality libraries with the Agilent 2100 Bioanalyzer, sequencing was performed on the Illumina Novaseq 6000 platform. For the analysis of differentially expressed miRNAs, the criteria used for filtering were a q-value < 0.05 and fold change (FC) > 2 or FC < 0.5. The DEG algorithm from the R package was employed to calculate q-values. Target gene prediction was carried out using the miranda software, with the parameters set as follows: S ≥ 150, ΔG ≤ -30 kcal/mol, and strict demand for 5’ seed pairing. Finally, differential expression miRNAs’ target genes were subjected to GO enrichment and KEGG pathway enrichment analyses using R packages. All small RNA sequencing and data analyses were performed by Eurofins Genomics (Shanghai, China).

### Animals

A total of 48 male Sprague-Dawley rats, aged 10 weeks, were generously provided by the Chinese Academy of Sciences Animal Center. Ethical approval for all animal experiments was obtained from the Wenzhou Medical University Animal Ethics Committee, with the approval number wydw2023-0355. The experimental groups were carefully designed as follows: the sham surgery group (undergoing only joint incision), the OA group (induced by anterior cruciate ligament transection and medial meniscus resection), the OA + MSCs-Exo group, and the OA + F-MSCs-Exo group, each comprising 6 rats [59]. In brief, starting from the fourth week after the establishment of the OA model, the OA + MSCs-Exo group and OA + F-MSCs-Exo group received intra-articular injections of MSCs-Exo or F-MSCs-Exo (10 μl/week), respectively [60]. The main aim of this study was to investigate and compare the therapeutic effects of MSCs-Exo and F-MSCs-Exo in the rat model of osteoarthritis.

To elucidate the role of miR-146b-5p in F-MSCs-Exo, the rats were further divided into four groups: the sham surgery group, the OA group, the OA + F-MSCs-Exo group, and the OA + F-MSCs-Exo + Antagomir-146b-5p group, with 6 rats in each group. In summary, starting from the fourth week after the establishment of the OA model, the rats in the OA + F-MSCs-Exo group received weekly intra-articular injections of F-MSCs-Exo (10 μl/week). Additionally, from the fourth week, the rats in the OA + F-MSCs-Exo group and the OA + F-MSCs-Exo + Antagomir-146b-5p group were treated with intra-articular injections of a negative control (nc) (5 nmol) or Antagomir-146b-5p (5 nmol) directly into the joint cavity. The experiment was conducted for a total of eight weeks post-surgery. At the end of the eight weeks, the rats were humanely euthanized under anesthesia, and knee joint samples were collected to assess the disease progression.

### Microcomputed tomography scans

Following the humane euthanization of the rats, knee joint specimens were carefully collected and preserved by overnight fixation in 4% paraformaldehyde. Subsequently, the fixed knee joint specimens underwent meticulous micro-computed tomography (micro-CT) using the advanced SkyScan-1276 micro-CT system (Bruker micro-CT, Kontich, Belgium). The micro-CT scans were conducted in three planes for each knee joint, encompassing the sagittal, transverse, and coronal planes. The acquired images were then employed to reconstruct the intricate 3D representation of the knee joint. Leveraging the 3D reconstructed images, comprehensive three-dimensional structural parameters were employed to meticulously evaluate the subchondral bone residing within the tibial plateau. This comprehensive evaluation facilitated a detailed and insightful analysis of both the cartilage and the underlying bone architecture within the knee joint.

### Histological analysis

After an 8-week duration, the knee joints of the rats were carefully harvested and fixed in 4% paraformaldehyde for 24 h. Following fixation, a 4-week decalcification process was carried out using a 10% EDTA solution (Solarbio, China). Subsequently, the tissues underwent meticulous processing, including dehydration, paraffin embedding, and sectioning. The obtained sections were then subjected to histological staining using Hematoxylin and Eosin (H-E) staining (Beyotime, China) as well as Safranin O-Fast Green (S-O) staining (Beyotime, China) for morphological analysis [61]. High-quality images of the stained sections were captured using an optical microscope. To comprehensively assess the condition of the cartilage, the internationally recognized Osteoarthritis Research Society International (OARSI) scoring system was employed [62]. This established scoring system enabled a detailed evaluation of structural changes in the cartilage and provided valuable insights into the extent of osteoarthritis pathology.

### Immunohistochemical analysis

The knee joints of the rats were fixed in 4% paraformaldehyde, followed by a series of processing steps, including decalcification, paraffin embedding, and sectioning. After dewaxing and dehydration, antigen retrieval was performed using 3% hydrogen peroxide. Subsequently, the sections were blocked with 10% goat serum (Solarbio, China) for 30 min at 4 °C and then incubated overnight at 4 °C with the primary antibodies (diluted at 1:200) for MMP-13, P62, and INOS. On the following day, the sections were incubated with HRP-conjugated secondary antibodies at 37 °C for 1 h [63]. Finally, visualization was carried out to examine the immunohistochemical analysis of the tissues.

### Statistical analysis

All data were expressed as mean ± standard deviation (SD) and analyzed using SPSS 20.0 software (Chicago, IL, USA). To identify intergroup differences, independent t-tests were performed, while one-way analysis of variance (ANOVA) was used for multiple group comparisons. Each experiment was conducted with a minimum of 3 biological replicates to ensure robustness and reliability. The significance levels were indicated as follows: *P < 0.05, **P < 0.01, and ***P < 0.001, representing the respective levels of statistical significance.

### Electronic supplementary material

Below is the link to the electronic supplementary material.

**Supplementary Material 1: Table.S1** The primers sequences used for qPCR. **Fig.S1** (A) Chemical formula of fucoidan. (B, C) 24, 48, 72 hours chondrocyte activity detected by CCK-8 method. **Fig.S2** (A, B)Quantitative analysis of Collagen II expression in rat knee joint tissue using immunofluorescent staining and ImageJ software (scale bar = 500 μm). **Fig.S3** (A) Gene Ontology (GO) analysis was performed on F-MSCs-Exo to investigate potential biological pathways and processes affected by these miRNAs in osteoarthritis treatment.

## Data Availability

Data will be made available on request.
