# Supplementary material for: MiR-146b-5p enriched bioinspired exosomes derived from fucoidan-directed induction mesenchymal stem cells protect chondrocytes in osteoarthritis by targeting TRAF6
Source: J Nanobiotechnology. 2023 Dec 18;21:486. doi: 10.1186/s12951-023-02264-9 (PMC10726686; doi:10.1186/s12951-023-02264-9)
Supplement: Supplementary file 1 — Supplementary Material 1: Table.S1 The primers sequences used for qPCR. Fig.S1 (A) Chemical formula of fucoidan. (B, C) 24, 48, 72 hours chondrocyte activity detected by CCK-8 method. Fig.S2 (A, B)Quantitative analysis of Collagen II expression in rat knee joint tissue using immunofluorescent staining and ImageJ software (scale bar = 500 μm). Fig.S3 (A) Gene Ontology (GO) analysis was performed on F-MSCs-Exo to investigate potential biological pathways and processes affected by these miRNAs in osteoarthritis treatment. [file 12951_2023_2264_MOESM1_ESM.doc]

**Supplementary Information**

**MiR-146b-5p Enriched bioinspired Exosomes Derived from Fucoidan-Directed Induction Mesenchymal Stem Cells Protect Chondrocytes in Osteoarthritis by Targeting TRAF6**

**Chao Lou^a,b.1^, Hongyi Jiang^a,b,1^, Zhongnan Lin^a,b^, Tian Xia^c^, Weidan Wang^a,b^, Chihao Lin^a,b^, Zhiguang Zhang^a,b^, Haonan Fu^a,b^, Shoaib Iqbal^d^, Haixiao Liu^a,b^, Jian Lin^a,b^, Jilong Wang^c,*^, Xiaoyun Pan^a,b,*^, Xinghe Xue^a,b,*^**

a Department of Orthopedics, The Second Affiliated Hospital and Yuying Children’s Hospital of Wenzhou Medical University, Wenzhou, Zhejiang Province, China

b Key Laboratory of Orthopedics of Zhejiang Province, Wenzhou, Zhejiang Province, China

c Wenzhou Institute, University of Chinese Academy of Sciences, Wenzhou, Zhejiang Province, China

d Feik School of Pharmacy, University of the Incarnate Word, 4301 Broadway, San Antonio, United States

* Corresponding authors:

Jilong Wang, E-mail: wangjilong@ucas.ac.cn

Xiaoyun Pan, E-mail: xiaoyunpan@126.com

Xinghe Xue, E-mail: 213205@wzhealth.com

Address: The Second Affiliated Hospital and Yuying Children’s Hospital of Wenzhou Medical University Wenzhou, Zhejiang, China.

1 These authors contributed equally to this work.

**Supplementary Table**

**Table.S1 The primers sequences used for qPCR**

| Genes | Forward primer sequence (5’-3’) | Reverse primer sequence (5’-3’) |
| --- | --- | --- |
| ATG7 | TGGAGCATGCCTACGATGAC | TTTGGGGTCCATACATCCGC |
| P62 | ACCCATCCACAGAGGCTGAT | GCCTTCATCCGAGAAACCCA |
| Beclin-1 | ACATCTGGCACAGTGGACAGTTTG | AGCATGGAGCAGCAACACAGTC |
| LC3 | GTCAGCGTCTCCACACCAATCTC | TCCTGGGAGGCATAGACCATGTAC |
| GAPDH | CAGGGCTGCCTTCTCTTGTG | GATGGTGATGGGTTTCCCGT |

**Supplementary Figure**


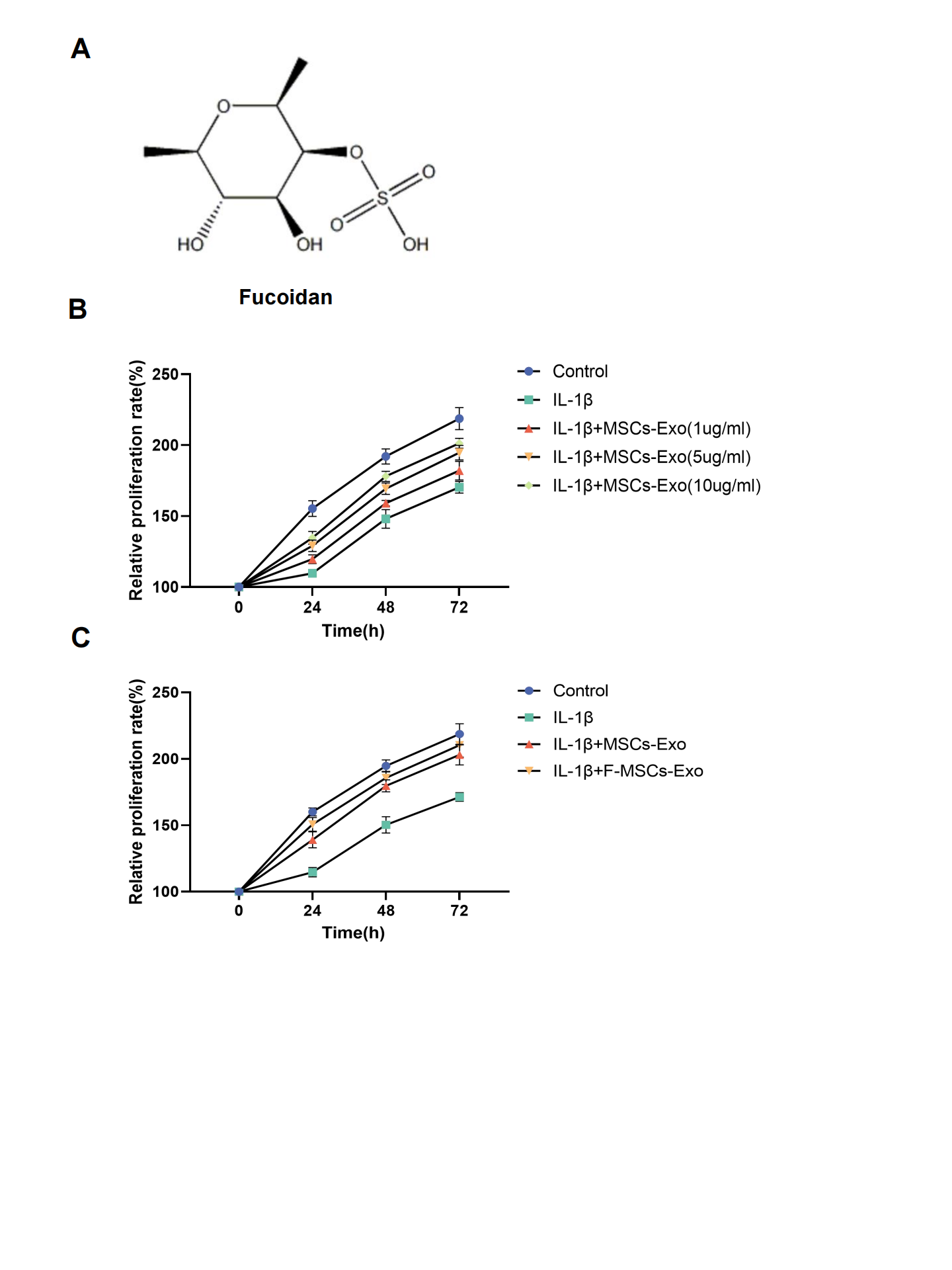


**Fig.S1** (A) Chemical formula of fucoidan. (B, C) 24, 48, 72 hours chondrocyte activity detected by CCK-8 method.


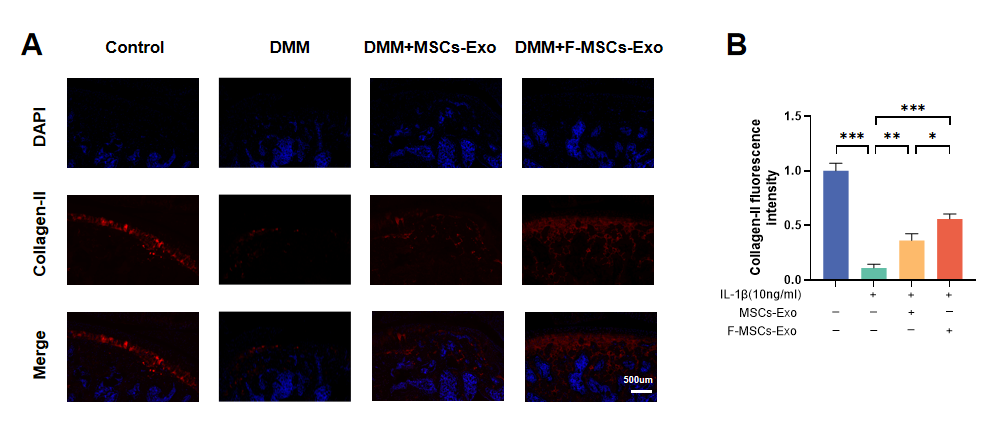


**Fig.S2** (A, B)Quantitative analysis of Collagen II expression in rat knee joint tissue using immunofluorescent staining and ImageJ software (scale bar = 500 μm)

**
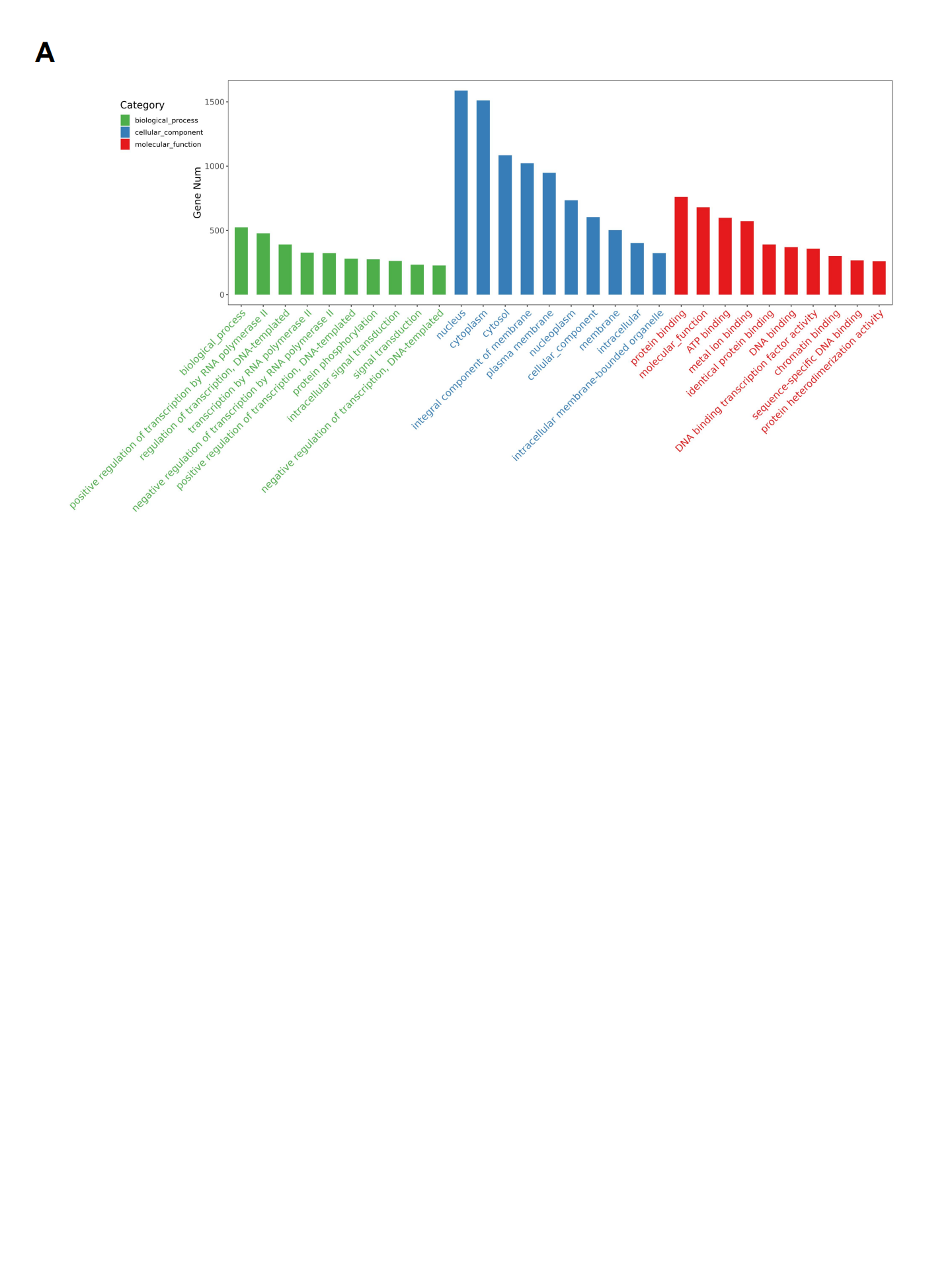
**

**Fig.S3** (A)Gene Ontology(GO) analysis was performed on F-MSCs-Exo to investigate potential biological pathways and processes affected by these miRNAs in osteoarthritis treatment.
